# ‘Talkin’ ‘Bout My Generation’: Using a Mixed-Methods Approach to Explore Changes in Adolescent Well-Being across Several European Countries

**DOI:** 10.3389/fpsyg.2017.00758

**Published:** 2017-05-18

**Authors:** Alina Cosma, Jelisaveta Belić, Ondřej Blecha, Friederike Fenski, Man Y. Lo, Filip Murár, Darija Petrovic, Maria T. Stella

**Affiliations:** ^1^Child and Adolescent Health Research Unit, School of Medicine, University of St AndrewsSt Andrews, United Kingdom; ^2^Department of Psychology, Faculty of Social and Behavioural Sciences, Leiden UniversityLeiden, Netherlands; ^3^Department of Psychology, Faculty of Arts, Masaryk UniversityBrno, Czechia; ^4^Department of Psychology, Faculty of Education and Psychology, Free University of BerlinBerlin, Germany; ^5^Division of Psychology and Language Sciences, Faculty of Brain Sciences, University College LondonLondon, United Kingdom; ^6^Department of Psychology, Faculty of Philosophy, University of Novi SadNovi Sad, Serbia; ^7^Department of Psychology and Cognitive Science, University of TrentoTrento, Italy

**Keywords:** adolescence, mental well-being, time trends, mixed methods study, HBSC

## Abstract

The promotion of positive mental health is a becoming priority worldwide. Despite all the efforts invested in preventive and curative work, it is estimated that one in four persons will experience a mental health condition at some point in their lives. Even more worrying is the fact that up to a half of all mental health problems have their onset before the age of 14. Recent statistics (national and international surveys, meta-analyses, international reports) point out to the fact that child and adolescent mental health problems are on the rise. The present study will try to corroborate these results and further explore their meaning, by employing a sequential mixed methods research design (quantitative–qualitative). The quantitative part will analyze time trends using Health Behaviors in School-aged Children data (four survey cycles: 2002, 2006, 2010, 2014) on mental well-being from four European countries (the Czechia, Germany, Italy, and United Kingdom). The qualitative part will rely on focus groups to explore the perspectives of 13- and 15-year-old boys and girls on gender differences and on the changes in adolescent mental well-being over time, as well as measures through which these issues could be addressed. Thematic analysis will be employed to analyze qualitative data. The results of this study could make a major contribution to our understanding of the current trends in adolescent mental well-being, as well as the ways in which existing data could be linked to international and national health policies.

## Introduction

The period of adolescence is fundamental for three reasons. Firstly, a healthy adolescence allows for the acquisition of certain developmental tasks, for example emotional and cognitive abilities; secondly, it can be viewed as the period for laying down health behaviors which determine the future health of such individuals; finally, adolescents will be parents of future generations so a healthy cohort of adolescence can lead to a future of healthy parents and children (Patton et al., 2016). All these premises are highly inter-related with mental health. For the purpose of this paper, the WHO conceptualization of mental health will be employed: “*a state of well-being in which the individual realized his or her own abilities, can cope with normal stresses of life, can work productively and fruitfully, and is able to make a contribution to his or her community*” (WHO, 2001).

There is a growing body of evidence showing that adolescence appears to be when many psychiatric disorders begin (Kessler et al., 2005). Mental health problems may influence a child’s learning and academic performance in school (Stoep et al., 2003) and have been linked to psychiatric disorders throughout adolescence and adulthood (Costello et al., 2003). Despite this, one in ten young people aged five to sixteen appear to experience a diagnosable mental health problem (Green et al., 2004), reflecting a need to pay greater attention to improving child and adolescent mental health.

Not only are a significant proportion of young people experiencing mental health problems, but, according to data from the HBSC study, it appears that mental well-being of adolescents also appears to be changing across cohorts. The HBSC collects cross-sectional data every four years on 11-, 13-, and 15-year-olds across more than 40 European and North American countries, measuring health and well-being, social environments, and health behaviors. Its advantage, therefore, is that it allows cross-cultural comparisons in data and a look at time trends in adolescent mental well-being. For example, using HBSC data from 35 European and North American countries, Ottová-Jordan et al. (2015) analyzed time trends (from 1994 to 2010) in mental well-being and found that in seven countries (Croatia, Greece, FYR Macedonia, Portugal, Slovenia, Spain, and Switzerland), there was a steady decline across cohorts; in five countries (Flemish Belgium, Denmark, Finland, Greenland, and Norway) a linear increase was found; in four countries (Austria, Canada, Czechia, and Scotland) a U-shaped trend was identified; in six countries (England, Estonia, Lithuania, Poland, Slovakia, and Sweden), an inverted U-shaped trend and unstable patterns in the remaining countries.

In a review that included studies from 12 countries, Bor et al. (2014) found that mental health symptoms in cohorts of children and toddlers generally improve or do not change. On the other hand, mental health trends in adolescents seem to be changing, and seemingly in a negative direction. There were no changes in terms of externalizing problems but evidence exists to suggest that internalizing problems are on the rise in cohorts of adolescents, particularly in adolescent girls. Parent-reported emotional problems in adolescents have also shown increases (Tick et al., 2007; Collishaw, 2015). Sweeting et al. (2010) collected data on a cohort of 15-year-olds in 1987 and compared it to a cohort in 2006, controlled for age, school year, and geographical location (West of Scotland). An increase in self-reported ‘psychological distress’ as measured on the General Health Questionnaire was found; this increase was significant for adolescent boys and girls but higher in girls. On the other hand, emotional problems in children appear to have decreased or not changed (Tick et al., 2007; Hölling et al., 2014; Matijasevich et al., 2014). Moreover, some studies used a qualitative approach to explore adolescents’ perspectives on well-being, yet it is difficult to find either cross-cultural qualitative studies in which adolescents are asked to interpret observed declining trends in well-being. A recent Navarro et al. (2015) suggests family relationships, peer to peer relationships, and school-related aspects to be the main topics adolescents refer to interpret reduced well-being. In a study on Spanish adolescents (Casas et al., 2012) physical safety, physical exercise, as well as life changes are emerging themes.

However, trends showing increasing mental health problems in adolescence may reflect either a genuine prevalence of emotional problems or increased reporting by adolescence, better recognition and reporting by parents and teachers, and better diagnoses techniques. Evidence suggesting that there is a genuine change comes from cross-cohort comparisons studies, which show increased self-reported depression and anxiety symptoms in adolescents since the 1980s in countries such as Greece, Germany, New Zealand Scotland, and England (Sweeting et al., 2009; Fleming et al., 2014; Collishaw, 2015). Similar studies in lower-income countries are lacking and would add to the amount of supporting evidence. Additionally, Collishaw (2015) reviews agreeing evidence from numerous study methods, informants and trends suggesting changes in some mental health problems, but not others, which would suggest genuine changes.

What new challenges are young people facing today that appear to be contributing to the deteriorating trends seen in adolescent mental health? There is yet to be a clear explanation; however, some possible explanations may include changes in individual vulnerability, socioeconomic and cultural factors, family life, and extrafamilial psychosocial factors (Collishaw, 2015). For example, there is evidence that the current generation of girls are experiencing puberty earlier than previous generations and this may coincide with a greater risk of developing depression (Bor et al., 2014). Moreover, social problems such as youth unemployment and economic recession may contribute to an increased risk of substance misuse and mental disorders in young people (Sawyer et al., 2012). Other studies have also found links between poverty and increased problems in adolescent mental health (Bradley and Corwyn, 2002; Costello et al., 2003; Reiss, 2013). Increasing rates of single parenting and other changes in the family environment may be contributory factors too (Bor et al., 2014). The increasing relevance that social media have in lives of young people may affect their mental well-being as well (Sawyer et al., 2012). Others suggest that lifestyle changes, for example increased sugar consumption, have accelerated rates of depression (Haroon et al., 2012).

Generational differences in adolescents are further complemented by gender differences where girls appear more likely to experience mental health issues than boys (Nolen-Hoeksema and Girgus, 1994; Salk et al., 2016). Possible explanations include differences in global self-esteem (Nolen-Hoeksema and Girgus, 1994), social media use (Monro and Huon, 2005), academic pressure (Byrne et al., 2007), or earlier sexualization, which may be related to worse self-esteem and depressed mood (Hatch, 2011). Many international and national policies, such as The European Mental Health Action Plan 2013–2020^1^, The European child and adolescent health strategy 2015–2020^2^, the Child and Adolescent Mental Health in Europe (CAMHEE) (Braddick et al., 2009), and the European Framework for Action on Mental Health and Wellbeing (Dahlgren and Whitehead, 2006) have been proposed to improve mental health and well-being in young people. However, for such targets to be fulfilled, understanding mental health in young people is essential to assuring that aims are met.

Thus, in this study, we aim to further assess the evidence that changes in cohorts of adolescents’ mental well-being are occurring and to gain an insight into the understanding of today’s adolescents on the reasons as to why adolescent mental well-being may be decreasing over time. To answer these research questions, this study will employ a mixed methods design (quantitative–qualitative). The quantitative part will aim to identify time trends in adolescent mental well-being across different European countries from 2002 to 2014. To do this, the quantitative part of the present study will rely on running secondary analysis of time trends using HBSC data from the United Kingdom (England, Wales, and Scotland), the Czechia, Germany, and Italy. Specific hypotheses which will be tested through the quantitative part will be the following:

(1) Based on previous findings, we predict that adolescent mental well-being indicators are decreasing across the cohorts in each of the examined countries.
(1) We expect to find a stable gender difference in mental well-being indicators with girls showing worse outcomes than boys.
(1) We expect to observe an age effect, the older cohort (15-year olds) will show worse outcomes than the younger cohort.

The qualitative part will explore young people’s perspective on the current trends in adolescent mental well-being (more specifically, the deteriorating mental well-being overtime). To explore these perceptions, series of focus groups with 13- and 15-year-olds will be run in the Czechia, Germany, England, Italy, and Serbia.

To summarize, the present mixed methods study will aim to answer the following two main research questions: *What are possible explanations for the observed trends indicating deteriorating adolescent mental well-being? And why are there gender differences in mental health trends?*

Current policies aiming to improve childhood and adolescent psychological health involve trying to identify indicators of poor psychological well-being at an earlier stage which may increase the success of treatment, increasing accessibility to mental health services for children and adolescents and reducing the stigma surrounding the topic of mental health. Ultimately, we hope that our findings will be able to impact public health policies around child and adolescent mental well-being, either by supporting the implementation of certain policies or revealing other areas of improvement.

## Materials and Methods

### Mixed Methods Design

**FIGURE 1:**
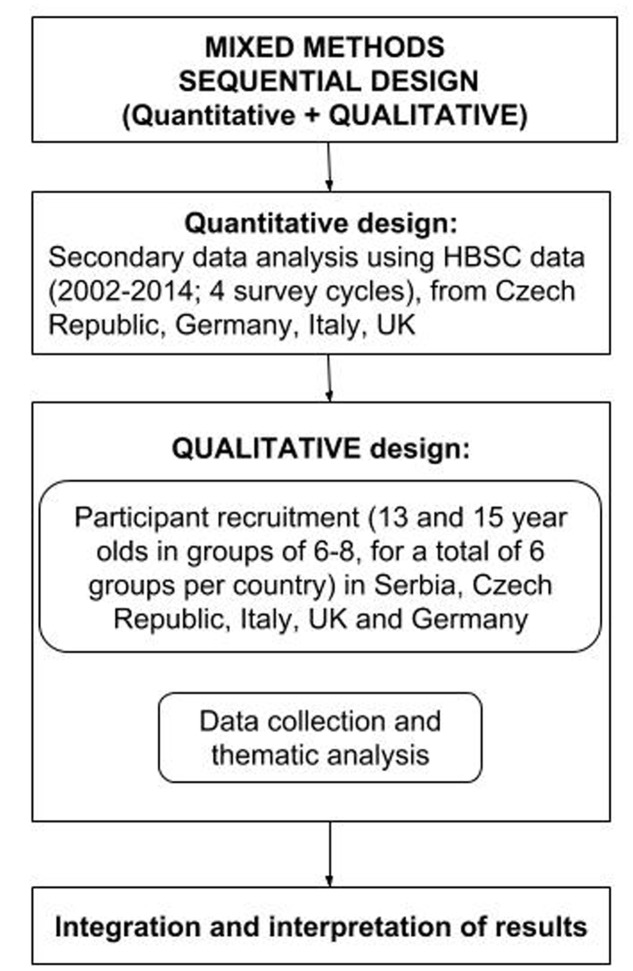
**The proposed research design**.

In the present study, a mixed methods approach will be employed, with a sequential quantitative–qualitative design (Tashakkori and Teddlie, 2003) (See **Figure 1**). A secondary analysis of quantitative data collected in the HBSC study^3^ (Inchley et al., 2016) will be performed to assess recent time trends in adolescent mental well-being across six European countries (England, Wales, Scotland, Czechia, Germany, and Italy). Qualitative data will be collected in focus groups with adolescent girls and boys to explore their perspective on the phenomena underlying changes in mental well-being and the challenges they experience. A sequential explanatory quantitative–qualitative design is needed since using quantitative data alone accurately shows emerging changes, but leaves us with limited practical guidelines relating to mental health policies. Therefore, applying a qualitative approach allows directly affected parties, in this case- adolescents, to become our main resource for themes which need to be addressed to understand observed changes in their mental well-being and to allow remedial interventions to be recommended.

### Psychosomatic Complaints

To analyze trends in mental well-being, the Psychosomatic Complaints measure of the HBSC will be used. This item consists of a series of questions where participants indicate the frequency with which they had experienced the following eight health complaints over the last six months; “headache”, “stomach ache”, “backache”, “feeling low”, “irritability or bad temper”, “feeling nervous”, “difficulties in getting to sleep” and “feeling dizzy”, (0 = “Rarely or never”, 1 = “About every month”, 2 = “About every week”, 3 = “More than once a week”, 4 = “About every day”). Responses across all eight complaints will be summed to generate a single score between 0 and 32, with higher values reflecting a greater psychosomatic complaint burden. This scale has undergone extensive qualitative and quantitative validation and shows good test-retest reliability and unidimensionality (Haugland et al., 2001). Reporting psychosomatic complaints is an important indicator for measuring subjective well-being, as it reflects personal experience related to negative life events in the social context of school, peers and family (Due et al., 2005; Hjern et al., 2008; Ottová-Jordan et al., 2015). They may also indicate a more serious underlying health problem (Ihlebæk et al., 2002). As opposed to some other well-being-related HBSC items, subjective health complaints are a sensitive measure (Ravens-Sieberer et al., 2009; Eriksson and Sellström, 2010), showing both individual-level (Ottová-Jordan et al., 2015) and international variation (Torsheim et al., 2006). Since somatic symptoms identified in late childhood and adolescence tend to persist into adulthood (Brattberg, 2004; Steinhausen and Winkler Metzke, 2007) and they may be predictive of somatization and anxiety symptoms in early adulthood (Kinnunen et al., 2010), psychosomatic symptoms in adolescence are an important indicator of mental health.

### Ethics Statement

For the secondary quantitative analysis, every HBSC participating country included in the present study (Czechia, Germany, England, Italy, Scotland, and Wales) has been given ethical clearance by national and regional governing bodies. For the qualitative part, ethical clearance will be obtained at national level by every research team member (Czechia, Germany, England, Italy, Serbia).

### Stepwise Procedure

We will conduct a mixed methods study with a sequential design which consists of the quantitative analysis followed by a qualitative investigation. Using secondary data analysis of already-collected survey data, the quantitative part will assess and describe changes in mental well-being of adolescents nowadays. It will be carried out by using the HBSC data from United Kingdom, Italy, Germany, and Czechia. The qualitative section of the present study will involve conducting focus groups with adolescents. This approach will allow for an in-depth exploration of possible explanations for the overall deteriorating trend of adolescent mental well-being. Tashakkori and Teddlie’s (2003) mixed methods sequential procedure will serve as a model for the methodological design for our study. One of the main assumptions of this procedure is using a quantitative approach to test theories, followed by a qualitative method that involves a detailed exploration with a few individuals. For example, Hodgkin (2008) used a sequential quantitative–QUALITATIVE approach to study women’s social capital. First, the author conducted a survey to identify different social capital profiles in the population. In a second step, Hodgkin (2008) used in-depth interviews to illuminate the stories behind these profiles with a few participants. The author argues, that the sequential approach allows to give a more powerful voice to groups that are not heard otherwise, in our case it will be the young people themselves.

### Quantitative Method

Data from four rounds of the international HBSC study will be used (2002, 2006, 2010, 2014). The HBSC study is a cross-sectional study of adolescent health carried out every four years across several European countries. At the moment, the HBSC network includes research teams from 45 countries across Europe and North America. Data collection is based on a standardized research protocol which specifies sampling methods and questionnaire content across all participating countries. For each survey round, countries collect a nationally representative sample of 11-, 13-, and 15-year-olds, with the timing of fieldwork arranged to achieve mean ages of 11.5, 13.5, and 15.5. Participants from each country were recruited via stratified random cluster sampling, with whole school classes as the sampling unit. Adolescents completed questionnaires in classroom settings, and could leave any question blank that they did not want to answer. Questionnaires were translated from English into respective national languages with back-translation checks. Appropriate ethical consent was gained in each participating country, with schools and adolescents giving active informed consent. For the current study, data will be collected from the Czechia, England, Germany, Italy, Scotland, and Wales, focusing on psychosomatic complaints. Responses across all eight complaints of the Psychosomatic Complaints item will be summed to generate a single score between 0 and 32 (with higher values reflecting a greater psychosomatic complaint burden), which will be used in a linear regression analysis.

### Qualitative Method

A qualitative approach in psychology is used mainly when a topic area needs to be further explored, for example when the investigated topic is complex, to empower individuals, to develop theories, and when mainstream quantitative measures simply do not fit the problem and do not provide us with meaningful information (Maxwell, 2009). In this study, by exploring the adolescents’ perspective on this issue, young people are identified as the most important and concerned party and their voices are empowered, making the qualitative approach appropriate for our aims. The qualitative data collection will be done through the use of focus groups. This method was selected by the research team due to its explorative nature (Sim and Snell, 1996). It enables the analysis of the attitudes, opinions and insights of adolescents on the research questions, to better understand the observed trends and give directions for future research.

Participants will be asked different questions regarding adolescence and mental health (please refer to Supplementary Material) to understand the adolescents’ perspective on mental well-being trends and the observed generational and gender differences in the trends. The focus groups will also provide us with some representation of what being an adolescent today consists of, including its positive and negative attributes.

Previous HBSC results showed a significant decrease in adolescent mental well-being indicators in both older age groups (13, and especially for the 15- years-olds). These two groups were chosen in the light of this information to be the participants of the focus groups. Their insights will allow us to improve our understanding of the challenges they are facing. The changes in mental well-being observed in 11- year- olds were largely negligible in the past research; therefore, this age group will not be included in the qualitative data collection. Participants in the qualitative part of the research will be 13- and 15- years-old schoolchildren in five participating countries – England, Germany, Czechia, Italy, and Serbia. A convenience sample from each country will be employed. The choice of the countries has been limited to these in particular due to the nature of the Junior Researcher Programme which represents the basic platform for the current study. The Junior Researcher Programme context in which the current study had its onset played the curtail role in the choice of the countries. The aim is to recruit a minimum of 36 adolescents per country. In order to achieve this, six focus groups with six to eight participants per country will be conducted. There will be three different focus group arrangements in both age groups: the single gender focus groups and mixed gender group. The reasoning behind this decision is based on the peculiarity of communication between boys and girls at this age, which may lead them to different answers while discussing the same topic in same-gender or mixed-gender setting.

The focus groups will be conducted in accordance with the focus group questions. This will be developed based on the latest theories and findings about adolescent mental well-being. Questions will originally be developed in English. In next phase the focus group guide will be translated to national languages (German, Italian, Czech, and Serbian) and back-translated to English. Prior to starting the data collection phase, a pilot study of the focus group guide will be conducted in every country. Through this, we aim to assess the overall flow of the questions, wording, question comprehension etc., before the finalization of the questions and the beginning of the field work. Before conducting the focus groups in each country, the recordings of the pilot interviews will be analyzed by the research team in order to ensure that the moderator has not influenced the participants’ in any way, such as asking question in a leading way. In addition, the questions developed for the discussion have been tailored to guarantee that the participants give their opinion on determined sub-topics of our interest, without having their views influenced by the moderator.

Once the participants have been recruited, the meetings will be arranged. Neutral locations are strongly preferred to avoid either positive or negative associations to the site or building. Focus group sessions will last approximately 60 to 90 minutes. At the beginning of every session, the moderator will provide a clear explanation of the purpose of the focus group. Once the purpose of the discussion is clear, participants will be introduced to the basic rules of conduct during the focus group discussions. Participants will be provided with an informed consent form, stating that they agree to take part in research. Within the consent, they will also be asked to agree with an audio-recording of the discussion.

Throughout the session, the moderator will initiate the discussion points and facilitate the exchange of ideas between participants. Depending on the group’s dynamics, moderators might need to promote debate, seek clearance, probe for details, or move the debate forward if the conversation loses its focus. Special attention will be given to ensure that each participant is given equal opportunity to speak. The moderators will be expected to avoid demonstration of personal opinions or any kind of preferences that could influence the participants’ train of thoughts. Participants will be informed of their valued position as experts on the matter at hand to motivate and empower them. They will also be informed of how unique this chance is to work collaboratively with the researchers and how they will be contributing to the greater good of their generation, and of those to come.

### Proposed Analyses

#### Analysis of Questionnaire Data

The analysis of questionnaire data will follow previous procedures used in other publications which used HBSC data (e.g., Whitehead et al., 2017). More specifically, questionnaire data will be stratified into subpopulations by country, and by gender and age within each country. Dataset weights, available with the raw HBSC dataset, will be applied in order to achieve national representativeness of each country at each time point. Linear regression analyses will be conducted using SPSS v.23 complex samples toolkit, which allows shared variance within sampling to be accounted for. The linear and quadratic effects of survey year on psychosomatic complaints will be evaluated for each subpopulation using general linear modeling.

#### Analysis of Focus Group Data

Qualitative data will be processed using thematic analysis, as defined by Braun and Clarke (2006). Initially, every author will read and re-read transcripts of recordings of the focus groups that they conducted in order to identify codes in the data, i.e., the basic features of the raw data that carry a meaning pertaining to the research questions (Braun and Clarke, 2006). Codes will be associated with quotes capturing the verbatim expression of the interviewed individuals. Analysis at this stage will be performed in the original languages, to minimize alteration or loss of meaning due to bulk translations. Once no more codes can be identified, the respective authors will sort the codes into semantically related categories, or themes. A set of candidate themes will then be repeatedly revised; initial themes may be split, merged or abandoned altogether while new themes may be formed. The aim will be to reach internal homogeneity and external heterogeneity of themes. As themes are being formed, they will be organized into a thematic map – a visual representation of the identified themes and their relationships – which may again be refined to achieve a map that validly and accurately reflects information contained in the data. In the next phase, codes, quotes and themes will be translated into English. Finally, these themes will be compared and collated to obtain a single thematic map capturing the insights from all the conducted focus groups. Similarities and differences between individual countries will be noted and the most representative quotes for each theme identified.

### Anticipated Results

The present study will contribute to the understanding of time trends in adolescent mental well-being across four European countries and offer possible explanations of their change. In contrast to previous studies, this research project will be based on an innovative sequential mixed methods design. This approach will allow for moving beyond the theoretical and statistical description of these time trends, as it will attempt to connect these results to young people’s interpretation of them. We expect that through this novel design (quantitative–qualitative), the findings could have a direct impact on future research, health policies, or interventions targeting adolescent mental well-being.

The quantitative design will allow us to identify the magnitude of change in adolescent mental well-being across the investigated countries. In line with previous studies, we firstly expect to find an overall decreasing trend of adolescent mental well-being between 2002 and 2014 across the investigated countries (for a review, see Bor et al., 2014). Furthermore, the countries are likely to differ in the characteristics of their respective time trends as shown in previous studies of mental well-being trends (Ottová-Jordan et al., 2015). Given that mental well-being also decreases with age, we also assume that, across all age groups included in the analyses, the 15-year-olds will show the greatest decline. This trend will be significantly different from both the 13-year-olds and the 11-year-olds (see, e.g., Ottová-Jordan et al., 2015). Finally, in line with results from the international HBSC report (Inchley et al., 2016), we expect to find gender differences, namely girls reporting more frequent psychosomatic complaints than boys across the investigated countries.

While the quantitative analysis will allow us to identify the extent of change in adolescent mental well-being across the investigated countries, the qualitative section of our study will give us the opportunity to directly ask young people about their opinion on the observed trends. Instead of reviewing possible explanations for them, our design will allow us to discuss actual challenges adolescents face with adolescents themselves. Thus, we expect the focus groups to be an environment that gives adolescents a voice to discuss perceptions, ideas, opinions, and thoughts about topics that are affecting their mental well-being. The aim of this process is to identify the factors underlying the observed deterioration in adolescent mental well-being in recent cohorts. Possible themes adolescents across participating countries may name are school pressure, the role of social media, or the role of family and friends (West and Sweeting, 2003; Collishaw, 2015; Patton et al., 2016). It is expected that instead of only offering possible explanations for the observed trends, adolescents may also provide possible solutions for tackling current adolescent mental well-being difficulties.

Several challenges in running the present study should be mentioned. Because focus groups in five countries will be conducted with five different official languages (Czech, German, Italian, Serbian, and English), it may be difficult to guarantee uniformity of all focus group guides regardless of the language they are written in. To ensure that questions with the same meaning will be asked across all countries, focus group guides originally developed in the English language will be translated to national languages and back-translated again to English. This process will be repeated until all focus group guides contain the same questions. Another challenge could lie in getting access to adolescents in different European countries in order to run the focus groups. Distinct EU Member States have different rules, regulations and guidelines regarding the participation of children in research projects, particularly concerning ethics approval and informed consent. Whilst some countries require only the parents’ informed consent (e.g., Czechia), other countries demand additional approval from respective supervisory school authority (e.g., Germany – consent from the federal state government). To ensure a smooth realization of the focus groups, researchers will make themselves familiar with the requirements of each respective country so that necessary documents can be prepared at an early stage. After successfully running the focus groups, an additional challenge will lie in the transcription of the data as well as the translation and retranslation of the transcripts to ensure that the data has been translated correctly.

Using a mixed methods approach allows a deeper understanding of the underlying processes regarding time trends in adolescent well-being. However, there are several limitations associated with the proposed study design. The main weakness of the design is the large amount of time involved in data collection (Creswell, 2009). Because HBSC survey data is being used, data collection is limited to the qualitative part only. However, the process of conducting the focus groups, the transcription of the audio-recorded focus groups, the translation and retranslation of the transcripts, and the analysis of the transcripts are very time-consuming. On the other hand, acquiring an in-depth insight into young people’s views on trends of mental well-being undoubtedly outweighs this limitation. Another frequent limitation of the qualitative designs is a lack of validity (Willig, 2008). It is critical that our focus group guide includes every important question to gain understanding of young people’s interpretation of time trends in adolescent mental well-being. To increase validity, pilot focus groups will be conducted where adolescents could provide feedback so that changes to our focus group guide can be made if necessary.

Many national and international policies have been proposed to improve mental well-being in young people, often co-founded under the EU Public Health Programs in recent years (Joint Action on Mental Health and Well-being, 2015). Most of these projects were implemented in schools, the elective setting for the prevention and/or promotion of the mental health of children and adolescents (e.g., CAMHEE, SEYLE, SCMHE, SUPREME). Each of the countries involved in our research has a national campaign for youth development and health. In the Czechia, The National Reference Centre of Programmes for Health Promotion and Disease Prevention supports programs focusing on stress coping, on children in crisis, and protection of children against violence (Jané-llopis et al., 2008). “MindMatters” is a program implemented in Germany that promotes mental health in primary and secondary schools (Braddick et al., 2009). The focus in Italy lies in the prevention of risk-taking behaviors through early identification of psychosocial stress, especially in young people, including interventions based on peer education and life skills education (Jané-llopis et al., 2008). The Strategy of Youth Development and Health in the Republic of Serbia 2007–2012 includes aims and activities to improve the quality, efficiency and accessibility of healthcare as well as to find new approaches for improving young people’s health (Expert Group on Youth Development and Health of the Ministry of Health of Serbia, 2006). In the United Kingdom, a Children and Young People’s Mental Health and Wellbeing Taskforce was set up in September 2014 to consider how to make it easier for children, young people, parents and carers to access help and support when they need it and to improve the help that is offered (Department of Health, 2015).

One of the first and most important priorities of the European Child and Adolescent Health Strategy 2015–2020 was to make adolescents’ lives more visible. We hope that our findings will add to this aim. Furthermore, we hope that they will highlight priority areas for action that can be used to inform the development and implementation of intervention and prevention programs. Overall, we anticipate that our results will contribute to the comprehension of how current adolescents feel and how they perceive themselves in their environments, as well as to connect these insights to European and national public health policies to counteract the continuous trend of deteriorating adolescent mental well-being around Europe.

## Author Contributions

This paper was created through collaborative activity and substantial intellectual contribution of all the authors listed above. AC proposed and developed the research idea, wrote parts of the manuscript, and coordinated the writing process. All the other authors wrote parts of the manuscript, provided important feedback when revising the manuscript. The publishing approval was given by all the authors.

## Conflict of Interest Statement

The authors declare that the research was conducted in the absence of any commercial or financial relationships that could be construed as a potential conflict of interest.

## Footnotes

1 “The European Mental Health Action Plan 2013–2020 – WHO/Europe” 2015. 24 Sep. 2016. <http://www.euro.who.int/__data/assets/pdf_file/0020/280604/WHO-Europe-Mental-Health-Acion-Plan-2013-2020.pdf>

2 “Investing in children: the European child and adolescent health…” 2014. 24 Sep. 2016 <http://www.euro.who.int/en/health-topics/Life-stages/child-and-adolescent-health/policy/investing-in-children-the-european-child-and-adolescent-health-strategy-20152020>

3 Open access is available the 2001/02, 2005/06, and 2009/10 HBSC datasets through the HBSC Data Management Center, based at the Norwegian Centre for Research Data (NSD). Because the datasets are restricted for the use of HBSC member country teams for a period of three years from survey completion, the 2013/2014 HBSC dataset is only scheduled for open access release by June 2018. However, aggregated datasets from the 2013/14 survey are available through the WHO Europe’s Health for All database (https://gateway.euro.who.int/en/data-sources/hbsc/). Further data is available for external use by agreement with the HBSC International Coordinator and the Principal Investigators.

## Abbreviations

EU: European Union
HBSC: Health Behaviours in School-aged Children
UK: United Kingdom
