# Supplementary material for: ‘Talkin’ ‘Bout My Generation’: Using a Mixed-Methods Approach to Explore Changes in Adolescent Well-Being across Several European Countries
Source: Front Psychol. 2017 May 18;8:758. doi: 10.3389/fpsyg.2017.00758 (PMC5436655; doi:10.3389/fpsyg.2017.00758)
Supplement: Supplementary file 1 [file Data_Sheet_1.docx]

# Appendix 1

Focus Group Guide

1. What does a life of an adolescent consist of today? What is iconic of being a teenager today? /*How is it like to be a teenager nowadays?/* What do you think about the mental well-being of adolescents these days?
2. What do you think mental well-being means? / How can we define mental well-being? / How does a mentally healthy person feel, behave?
3. (*Presenting graph on trends)* What are your thoughts on why this may be happening? /*How would you explain this development?/* Why do you think we see this trend? What can be the cause behind this trend? *How (and thus why) do you think your generation differs from prior generations?*
4. *Why are young people nowadays more likely to indicate worse mental well-being compared to young people 14 years ago?/* What are the main challenges/problems adolescents are facing/having?/ *What is special about 15/yo that makes them more vulnerable (or more likely toreport lower mental well-being?)*
   1. Do you think it's the environment that adolescents are growing up in that's different, or the way adolescents are taught to handle challenges in life?
   2. Do you think that social media is having an impact on your mental well-being and why is it so?
5. Why are there gender differences?/ *How are adolescent boys and adolescent girls different?* /What do you think are the reasons for the different trends for boys and girls? What’s different for both genders?/ *Are the challenges same for both genders?*/ What are (15yo) girls facing that boys don’t? Why are 15 yo girls more likely to report lower mental well-being?
6. Of all the things we have discussed, what is in your opinion the most important reason for the change?
7. Talking about this trends, is there something you would like to emphasise/add?/ *Does anyone else have anything to say about generations differences?*
8. Would you please think about what needs to happen so that your mental well-being improves? *What do you think could be done in order to make the adolescents more satisfied?*/ What kind of support do you think would be of use to adolescents’ in order to change this trend? [Are you getting that kind of support? If not, what is stopping you from having it?]
   1. What could the school/parents/society change?
9. What are some positive things about being 13/15 today?/ *What do you like best of being an adolescent?*
